# Evaluating the CARE4Carer Blended Care Intervention for Partners of Patients With Acquired Brain Injury: Protocol for a Randomized Controlled Trial

**DOI:** 10.2196/resprot.9108

**Published:** 2018-02-16

**Authors:** Vincent CM Cox, Vera PM Schepers, Marjolijn Ketelaar, Caroline M van Heugten, Johanna MA Visser-Meily

**Affiliations:** ^1^ Center of Excellence in Rehabilitation Medicine, Brain Center Rudolf Magnus University Medical Center Utrecht Utrecht University and De Hoogstraat Rehabilitation Utrecht Netherlands; ^2^ Department of Rehabilitation, Physical Therapy Science & Sports Brain Center Rudolf Magnus University Medical Center Utrecht, Utrecht University Utrecht Netherlands; ^3^ Department of Neuropsychology and Psychopharmacology Faculty of Psychology and Neuroscience Maastricht University Maastricht Netherlands; ^4^ School for Mental Health and Neuroscience Faculty of Health, Medicine and Life Sciences Maastricht University Medical Center Maastricht Netherlands; ^5^ Limburg Center for Brain Injury Maastricht Netherlands

**Keywords:** caregivers, brain injuries, internet, telemedicine, randomized controlled trial

## Abstract

**Background:**

Support programs for partners of patients with acquired brain injury are necessary since these partners experience several unfavorable consequences of caregiving, such as a high burden, emotional distress, and poor quality of life. Evidence-based support strategies that can be included in these support programs are psychoeducation, skill building, problem solving, and improving feelings of mastery. A promising approach would seem to be to combine web-based support with face-to-face consultations, creating a blended care intervention.

**Objective:**

This paper outlines the protocol of a randomized controlled trial to evaluate the CARE4Carer blended care intervention for partners of patients with acquired brain injury.

**Methods:**

A multicenter two-arm randomized controlled trial will be conducted. A total of 120 partners of patients with acquired brain injury will be recruited from five rehabilitation centers in the Netherlands. The blended care intervention consists of a nine-session web-based support program and two face-to-face consultations with a social worker. Themes that will be addressed are: giving partners insight into their own situation, including possible pitfalls and strengths, learning how to cope with the situation, getting a grip on thoughts and feelings, finding a better balance in the care for the patient with acquired brain injury, thinking about other possible care options, taking care of oneself, and communication. The intervention lasts 20 weeks and the control group will receive usual care. The outcome measures will be assessed at baseline and at 24- and 40-week follow-up. The primary outcome is caregiver mastery. Secondary outcome measures are strain, burden, family functioning, emotional functioning, coping, quality of life, participation, and social network.

**Results:**

The effect of the intervention on the primary and secondary outcome measures will be determined. Additional a process evaluation will be conducted.

**Conclusions:**

The findings of this study will be used to improve the care for partners of patients with acquired brain injury. Barriers and facilitators that emerge from the process evaluation will be used in the nationwide implementation of the intervention.

**Trial Registration:**

Dutch Trial Register NTR6197; http://www.trialregister.nl/trialreg/admin/rctview.asp?TC=6197 (Archived by WebCite at http://www.webcitation.org/6xHBAxx0y)

## Introduction

Caregivers of patients with acquired brain injury (ABI), such as stroke and traumatic brain injury, often experience high levels of burden, which profoundly affects their physical and psychosocial well-being [1-5]. About half of the caregivers experience anxiety and emotional distress, and 65% report health problems and a decline in social life with high levels of strain [6]. The majority of caregivers of patients with ABI reporting psychological distress are the partners of the patients [7]. Among partners of patients who were admitted for inpatient rehabilitation, 80% reported poor quality of life one year after stroke [3]. It is especially the return home after inpatient rehabilitation which appears to be a major hurdle for patients and their caregivers [6].

Support programs for partners of patients with ABI are necessary and should be initiated as early as possible after discharge from the rehabilitation facility so partners are better prepared for their new role as caregivers at home [8]. Several reviews show that evidence-based support strategies such as psychoeducation, problem-solving therapy and skill building are effective components of interventions [9-13]. Additionally, support programs should address condition-specific issues, such as the cognitive, emotional, and personality changes of the patient [1]. Furthermore, interventions to increase feelings of mastery also seem important, since mastery can protect against the stressors of caregiving and improve caregivers’ well-being [14,15].

Participating in a support program can be challenging for partners of patients with ABI, since being a caregiver already takes up much time and energy [16], in addition to everyday activities such as having a job. Travelling to a rehabilitation center to attend a support program can be experienced as requiring too much time and energy. Web-based interventions may therefore be more suitable, since partners can participate at any time from any location with internet access, and they can keep their own pace [17]. Caregivers in various populations have reported being satisfied and comfortable with web-based interventions [18]. Previous research has shown that web-based interventions can improve family functioning, psychological well-being, coping, and quality of life among caregivers [18,19]. Furthermore, web-based interventions for caregivers are feasible [19] and can save costs [18]. A disadvantage of web-based interventions, however, is that of the higher drop-out rates [20]. Participants of web-based interventions report that adherence can be increased by combining web-based interventions with face-to-face consultations, creating a blended care intervention [21,22]. Another advantage of combining web-based interventions with face-to-face consultations is the opportunity for personalized treatment, elaborating on specific personal problems which cannot be addressed through predefined responses but require input from professional caregivers [22].

This study aims to evaluate the effects and process of a blended care intervention, which includes psychoeducation, skill building and problem solving, on feelings of mastery in partners of patients with ABI. Our hypothesis is that the intervention group will have increased feelings of mastery compared to the control group. This paper describes the study protocol.

## Methods

### Design

This study is a multicenter two-arm randomized controlled trial investigating the CARE4Carer blended care intervention in addition to usual care, in comparison to usual care alone. The Medical Research Ethics Committee of the UMC Utrecht confirmed that the Dutch Medical Research Involving Human Subjects Act (WMO) does not apply to this study. The Dutch Agreement on Medical Treatment Act (WGBO) and Dutch Personal Data Protection Act (Wbp) do apply. All participating rehabilitation centers have approved the study protocol. Written informed consent is obtained from each participant. The study is registered in the Dutch trial register as NTR6197, registered 2 November 2016.

### Participants

The study population consists of partners of patients with ABIs such as stroke, subarachnoid hemorrhage, traumatic brain injury, postanoxic encephalopathy (ie, acute onset, no degenerative neurological diseases). Participants are recruited from five rehabilitation centers in the Netherlands (Adelante, Heliomare, Reade, Sint Maartenskliniek, Tolbrug). Inclusion criteria for both patient and partner are: (1) 18 years or older, and (2) written informed consent. Additional inclusion criteria for the patient are: (1) having an ABI, (2) independent living in the community before the ABI, (3) having been admitted for inpatient rehabilitation, and (4) being scheduled to be discharged home after rehabilitation. Additional inclusion criteria for the partner are: (1) being one of the patient’s primary caregivers, and (2) being the patient’s partner. Exclusion criteria for the patient are: (1) neurodegenerative or progressive ABI and (2) insufficient command of Dutch, clinically judged by the health care professionals. Exclusion criteria for the partner are: (1) insufficient command of Dutch, clinically judged by the health care professionals, (2) being unable to work on a computer, and (3) having no internet access. Partners can only participate when the patient signs informed consent. If the patient decides to stop study participation, the partner can continue to participate, but data of the patient will not be used in the analyses.

### Procedure

The participants are recruited during regular consultations with a social worker during inpatient rehabilitation. The social workers, who are trained in the treatment protocol, check the eligibility criteria and explain the study. Both partner and patient receive an information letter and an informed consent form and are asked whether the researcher may contact them. After a few days, the researcher calls the partner and asks if there are any questions about the study. When both partner and patient agree to participate, they sign their informed consent forms and return these to the researcher by mail. Reasons for exclusion and reasons to decline research participation are recorded. Randomization takes place after the informed consent forms have been received.

**Figure 1:**
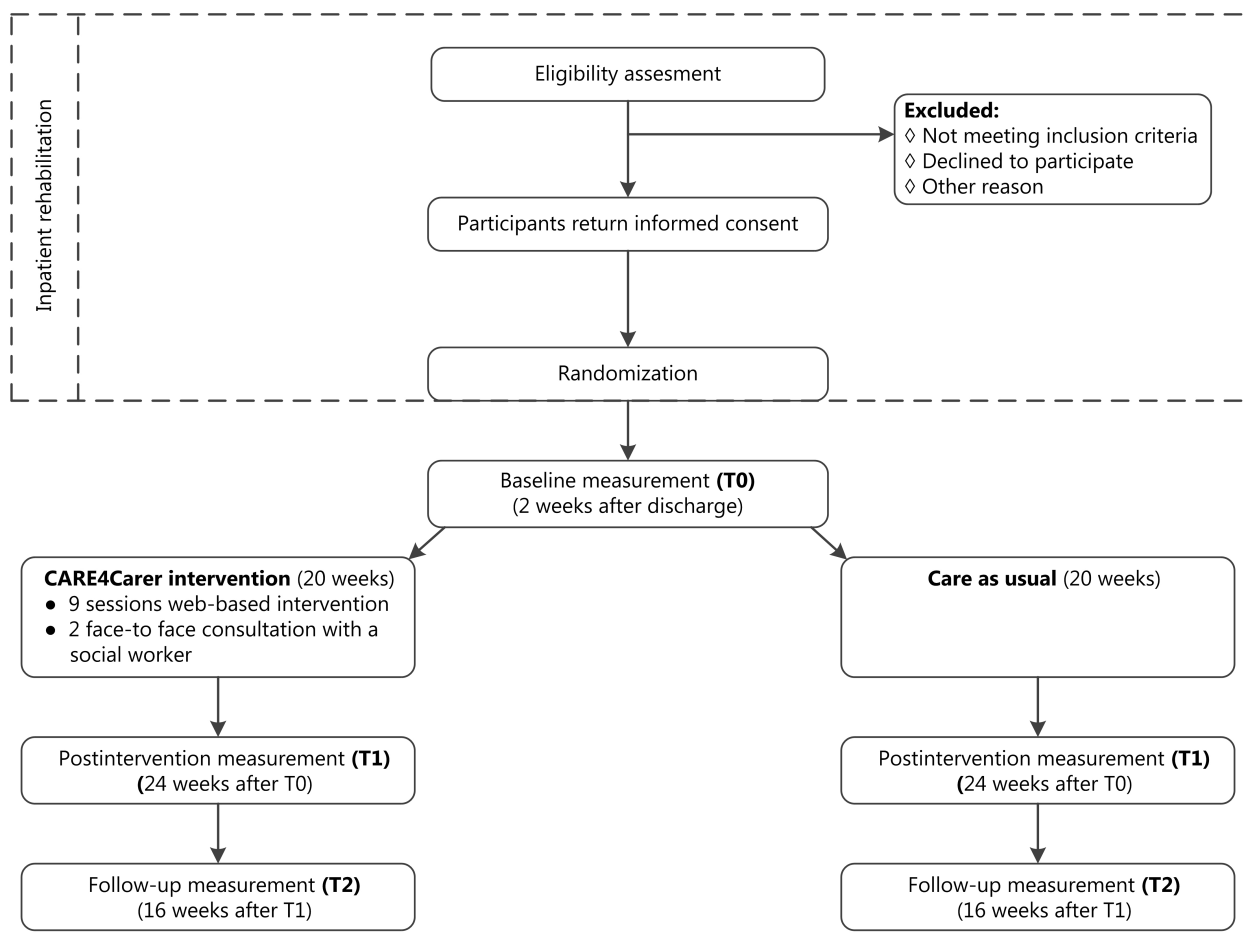
Flow diagram of the trial.

Demographic factors of the partners are recorded at baseline and those of the patients are retrieved from the patient records. The outcome measures for the partners are assessed at baseline, postintervention and at follow-up, except for care consumption, which is not assessed at baseline. The outcome measures for the patients are assessed at baseline and at follow-up. Questions regarding process evaluation are presented after the intervention. All questionnaires are administered through the same platform, which also provides the web-based support program. See Figure 1 for the flow diagram.

### Randomization

Participants are randomly assigned to either the group receiving the CARE4Carer intervention or to the usual care control group, using an online randomization tool **.** Participants are stratified by rehabilitation center, and block randomization with two block sizes (2 and 4) is used to achieve a balance across the experimental and control groups. The block size and order of allocation are randomly chosen at the beginning of each block. This minimizes the risk of predicting group assignment and keeps the researcher blinded to the randomization process. Randomization takes place before the baseline measurement to be able to assign a certain route to the partner in the online platform. Partners in the intervention group automatically gain access to the web-based support program directly after completing the baseline measurement, which is only possible when this route is set beforehand.

### CARE4Carer Intervention

The CARE4Carer intervention starts two weeks after the patient is discharged from inpatient rehabilitation and consists of a web-based support program and face-to-face consultations with a social worker.

### Web-Based Support Program

The intervention program, called Brain injury – Moving forward together (in Dutch: “Hersenletsel – hoe samen verder?”), is a web-based support program for partners of patients with ABI. The program comprises 9 sessions, described in Textbox 1. It is based on an existing support program, which was developed by Minddistrict and Heliomare Rehabilitation Centre. They used principles of cognitive behavior theory [23] and solution-focused therapy [24], as well as expert input from social workers, psychologists, and caregivers of patients with ABI.

We have modified this program to tailor it specific to partners of patients with ABI. We have also carried out a pilot study in which three partners of patients with ABI and a member of the patient association tested and evaluated the program. This has led to several further adaptations. Themes within the program are: getting insight into one’s own situation, including possible pitfalls and strengths, learning how to cope with the situation, getting a grip on thoughts and feelings, finding a better balance in the care for the patient with ABI, thinking about other possible care options, taking care of oneself, and communication. Each session is informative and easy to use and provides practical tips. The sessions consist of psychoeducation and assignments aimed at problem solving and skill building. Short videos featuring a social worker and videos of partners who are caregivers of patients with ABI are included in each session.

The partners can attend the program over a period of 20 weeks in their own time, at their own pace, and from any location with internet access. Partners are encouraged by automatic email reminders and by the social workers to complete the sessions before the postintervention measurement, although the program is still available for them after this period.

### Face-to-Face Consultations

In addition to the web-based support program, partners are offered two consultations with a social worker at the rehabilitation center. The social workers prepare for the meetings by reviewing the completed assignments presented in the web-based sessions. Issues emerging from these answers are addressed and specific personal situations are discussed. The first consultation takes place 10 weeks after discharged, after the first 4 sessions of the web-based program have been completed; the second consultation is after the 9^th^ session, 20 weeks after discharge. The duration of the consultations is about 45 minutes to one hour, depending on the need to elaborate.

### Usual Care

Partners randomized to the control group receive usual care. This can consist of consultations with a social worker and/or psychologist and peer support groups. Partners in the intervention group are also allowed to receive usual care in addition to the CARE4Carer intervention.

### Measures

The primary outcome is caregiver mastery. Secondary outcome measures for the partners are strain, burden, family functioning, emotional functioning, coping, care-related quality of life, participation, social network and care consumption. Secondary outcome measures for the patients are family functioning, emotional functioning, and participation. Additionally, a process evaluation will be conducted. An overview of all instruments and the time of assessment is presented in Table 1.

### Caregiver Mastery

Caregiver mastery is measured by the Caregiver Mastery Scale (CMS) [25]. This instrument is an adaptation of the Pearlin Mastery Scale [26], in order to measure mastery in the caregiving situation instead of global mastery. This questionnaire consists of seven statements about caregiving, such as “You believe you are mastering most of the challenges in caregiving.” Partners are asked to indicate their level of agreement (ie, strongly disagree, disagree, neither agree nor disagree, agree, strongly agree) with each statement. Three items with negative statements are reverse-scored. Total scores can range from 7 to 35, with higher scores reflecting greater caregiver mastery. Psychometric quality has been confirmed [25] and the instrument has proved to be able to detect change after intervention [27].

### Secondary Outcome Measures

#### Caregiver Strain Index (CSI)

The amount of strain experienced by the partner is assessed with the CSI. This instrument contains 13 statements which are scored a 1 (“yes”) or 0 (“no”) [28]. Total scores range from 0 to 13, with higher scores indicating higher strain. Scores of 7 or higher indicate substantial strain. The CSI is a reliable [28] and valid [29] instrument which is commonly used for caregivers of stroke patients [30].

#### Self-Rated Burden (SRB)

A single question enables the partners to indicate how burdensome caring for the patient with ABI is at that moment. A visual analogue scale is used, ranging from 0 (“not hard at all”) to 100 (“much too hard”) [29]. The SRB has proved to be a valid instrument to assess the burden of caregiving for informal caregivers of patients with stroke [29].

**Sessions of the CARE4Carer web-based intervention.**

1. Welcome
2. Caring for your partner
3. Burden and resilience
4. Which care choices to make?
5. Getting a grip on your thoughts and feelings
6. Taking care of yourself
7. Asking for support
8. Communication
9. And now?

**Table 1:** Overview of all instruments.

Instruments	T0		T1		T2	
	C **^a^**	P **^b^**	C	P	C	P
Caregiver Mastery Scale	x		x		x	
Caregiver Strain Index	x		x		x	
Self-Rated Burden	x		x		x	
McMaster Family Assessment Device Subscale: General Functioning	x	x	x		x	x
Hospital Anxiety and Depression Scale	x	x	x		x	x
Utrecht Coping List	x		x		x	
CarerQol	x		x		x	
Utrecht Scale for Evaluation of Rehabilitation – Participation Subscale: Restrictions	x	x	x		x	x
Social network	x		x		x	
Care consumption			x		x	
Process evaluation			x			

^a^C=caregiving partner.

^b^P=patient.

#### McMaster Family Assessment Device (FAD)

Family functioning is assessed with the General Functioning subscale of the FAD [31]. Partners indicate their level of agreement (ie, strongly disagree, disagree, agree, strongly agree) with 12 statements. Each statement is scored from 1 to 4, with 1 reflecting healthy functioning and 4 reflecting unhealthy functioning. A mean score of 2.0 or higher indicates problematic family functioning [32]. The FAD has good psychometric properties [31-33].

#### Hospital Anxiety and Depression Scale (HADS)

Emotional functioning is measured with the HADS. It consists of two 7-item subscales measuring anxiety and depression. Scores above 7 on the subscales indicate an anxiety disorder or depression, respectively [34]. The HADS has good psychometric properties and has proved to be responsive to change [35,36].

#### Utrecht Coping List (UCL)

Coping is assessed with three subscales of the Utrecht Coping List: (1) active problem solving (7 items), (2) seeking social support (6 items), and (3) passive reacting (7 items) [37]. A 4-point rating scale is used, ranging from “seldom or never” to “very often”. Higher scores on a subscale indicate a greater tendency to use that specific coping style. The UCL has good psychometric properties [37,38].

#### CarerQol

The CarerQoL instrument measures the care-related quality of life of informal caregivers [39]. It determines the subjective burden in seven dimensions of the caregiving situation (CarerQol-7D) and includes a valuation component (CarerQol-VAS). Low scores on the CarerQol-7D indicate a poor caregiving situation, while high scores on the CarerQol-VAS reflect a higher level of happiness. The CarerQol is a valid tool to measure the impact of caregiving [40].

#### Utrecht Scale for Evaluation of Rehabilitation – Participation (USER-P)

Participation restrictions are assessed with the USER-P instrument [41]. On 10 items, respondents indicate to what extent they are able to do the activity described. Scores range from 0 (“not possible”) to 3 (“without difficulty”). Higher total scores indicate fewer participation restrictions. The USER-P has good psychometric properties [42,43].

#### Social network

The social network (ie, number of parents/step-parents, children/grandchildren, other family members, and friends/neighbors) is mapped using a newly developed questionnaire. It also includes items about how easy it is to get practical and emotional help from these persons. Partners answer on a 5-point scale ranging from "very easy" to "very difficult".

#### Care consumption

Care consumption is assessed during the post-intervention (T1) and follow-up (T2) measurements. Partners are asked whether and how often they have had contact with a psychologist, social worker, general practitioner, practice nurse and/or aftercare nurse, and whether they participated in peer support groups.

### Process Evaluation

At postintervention (T1), the partners evaluate the intervention, the individual sessions, and the different elements of the intervention by filling in the online questionnaire. The advantages, disadvantages, satisfaction, and usability of the intervention are investigated.

Using interviews, we assess the experiences of the social workers with carrying out the intervention and working with a blended care program, as well as their views on facilitators and barriers for implementation. Every social worker who supported a caregiver in the intervention group will be interviewed.

Treatment fidelity is determined by reports from the social workers on the number of face-to-face consultations that have taken place and by analyzing how many sessions of the web-based support program have been completed. Partners are obliged to fill in certain assignments to be able to complete a session.

### Blinding

The baseline measurements (T0) are self-reported by partner and patient, who do not yet know the allocation outcome at this stage. Blinding to treatment allocation is not possible due to the nature of the intervention. The postintervention (T1) and follow-up (T2) measurements are, therefore, not blinded since these are self-reported by the partner and patient who are aware of treatment allocation by that time.

### Power Analysis

The sample size has been calculated on the basis of the primary outcome measure, the Caregiver Mastery Scale. To detect a difference between the groups of 0.5 SD on the Caregiver Mastery Scale, with an alpha of 0.05 and a power of 80%, a total of 50 caregivers is needed in each arm of the trial. Assuming a drop-out rate of 20%, a total of 120 patient-partner couples will be included in the CARE4Carer trial.

### Statistical Analyses

Descriptive statistics including frequencies, means, standard deviations, and (for nonparametric data) medians and interquartile ranges will be calculated. Longitudinal data analysis will be performed using a generalized linear mixed model (GLMM), to evaluate differences in efficacy between the experimental and control groups. Data will be analyzed based on an “intention-to-treat” analysis and with an alpha level of 0.05. The analysis software IBM SPSS Statistics version 22 for Windows will be used [44]. Descriptive statistics will be used for the partners’ process evaluation and for the treatment fidelity. The interviews with the social workers will be transcribed verbatim and qualitative analyses will be performed.

## Results

Participant recruitment for this randomized controlled trial commenced in September 2016 and enrolment is ongoing. The first results are expected to be submitted for publication in 2018.

## Discussion

In this paper, we have described the protocol of a randomized controlled trial to evaluate the CARE4Carer blended care intervention to improve feelings of mastery in partners of patients with acquired brain injury. We will also investigate the effect of the intervention on strain, burden, family functioning, emotional functioning, coping, quality of life, participation, social network, and care consumption. A process evaluation will also be part of this study.

*Brain injury – Moving forward together* is an innovative partner support program. It was developed in cocreation with partners, social workers, and psychologists. Methods that have proved to be effective, such as those based on cognitive behavior theory and solution-focused therapy, have been integrated in the intervention. The program has been pilot-tested among partners of patients with ABI and modified in response to their comments. Another strength of this study is the use of blended care. Integrating the web-based support program with face-to-face therapy combines the best of two worlds, which can enhance the effect of the intervention [22]. To our knowledge, this is the first blended care intervention for partners of patients with ABI.

It is important to note that blended care is probably not suitable for everyone. Not every partner and health care provider may be ready for blended care. Some partners may not be comfortable with receiving support via a web-based program and might prefer to only have face-to-face contacts. Also, health care providers may resist offering support via the internet [45]. In addition, access to internet and possession of a computer, laptop, tablet or smartphone is not standard for everybody; 22% of the Dutch population aged 65 years or older has no internet access at home [46].

The study may have some limitations. First, we only include partners of patients who are admitted for inpatient rehabilitation. Patients who go home after treatment at the hospital and patients who receive geriatric rehabilitation are not included. Second, the control group treatment is not standardized, because care as usual differs between rehabilitation centers.

Support for partners of patients with ABI is clearly needed. Blended care interventions that include psychoeducation, skill building and problem solving have not been investigated in this population yet. Our CARE4Carer intervention could help partners to better deal with their new role as a caregiver, after the patient has returned home. We hypothesize increased caregiver mastery among partners as a result of this intervention. The findings of this study will be used to inform rehabilitation physicians, social workers, and psychologists and to improve the care for partners of patients with ABI. If the intervention proves to be superior to usual care, it will be made available for implementation nationwide, taking into account the barriers and facilitators that emerge from the process evaluation.

## Abbreviations

ABI: acquired brain injury
CarerQol-7D: care-related quality of life – 7 dimensions of subjective burden
CarerQol-VAS: care-related quality of life – Visual Analogue Scale
CMS: Caregiver Mastery Scale
CSI: Caregiver Strain Index
FAD: Family Assessment Device
GLMM: generalized linear mixed model
HADS: Hospital Anxiety and Depression Scale
SRB: Self-Rated Burden
UCL: Utrecht Coping List
USER-P: Utrecht Scale for Evaluation of Rehabilitation – Participation
Wbp: Wet bescherming persoonsgegevens (Dutch Personal Data Protection Act)
WGBO: Wet op de geneeskundige behandelingsovereenkomst (Dutch Agreement on Medical Treatment Act)
WMO: Wet medisch-wetenschappelijk onderzoek met mensen (Dutch Medical Research Involving Human Subjects Act)

## Appendix

Multimedia Appendix 1 Screenshot overview of web-based support program.

Multimedia Appendix 2 Screenshot session 3: burden and resilience.
