# Supplementary figures and images for: Evaluating the CARE4Carer Blended Care Intervention for Partners of Patients With Acquired Brain Injury: Protocol for a Randomized Controlled Trial
Source: JMIR Res Protoc. 2018 Feb 16;7(2):e60. doi: 10.2196/resprot.9108 (PMC5834754; doi:10.2196/resprot.9108)

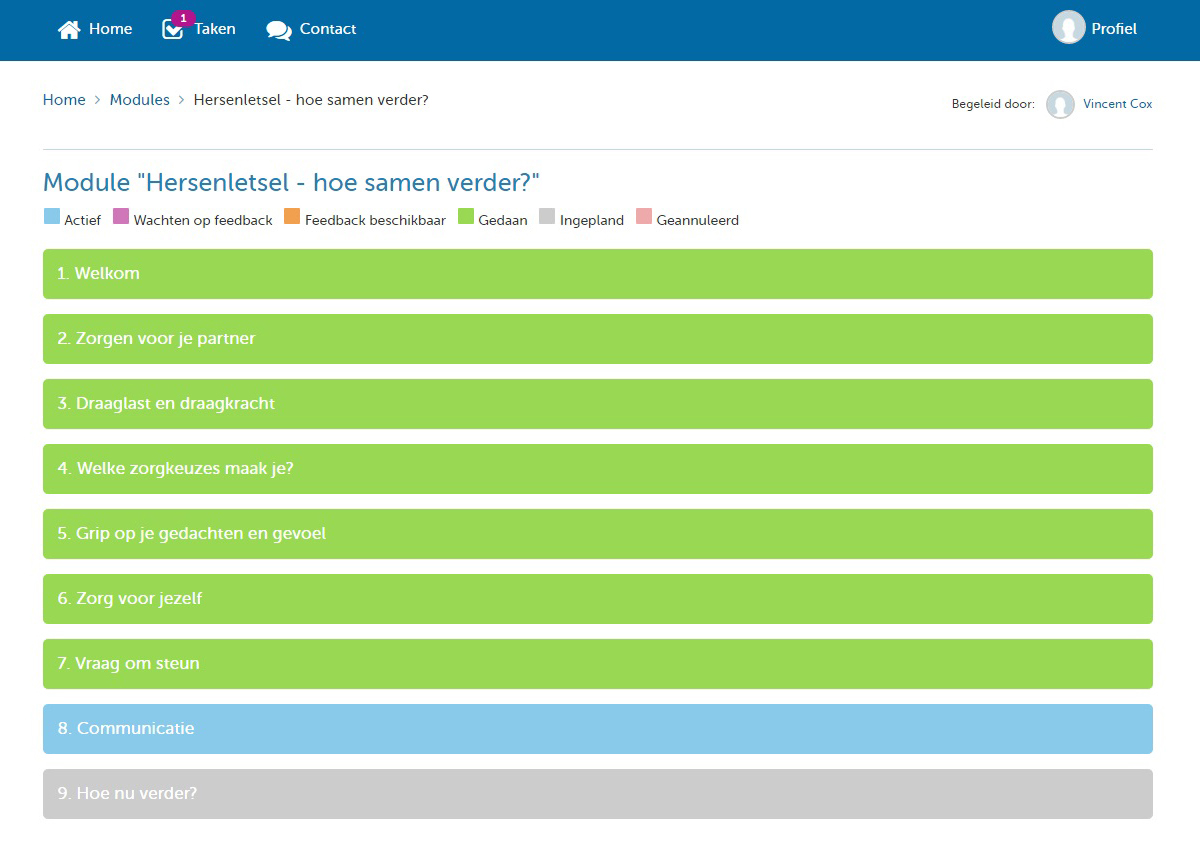

Supplement: Multimedia Appendix 1 [file resprot_v7i2e60_app1.jpg]

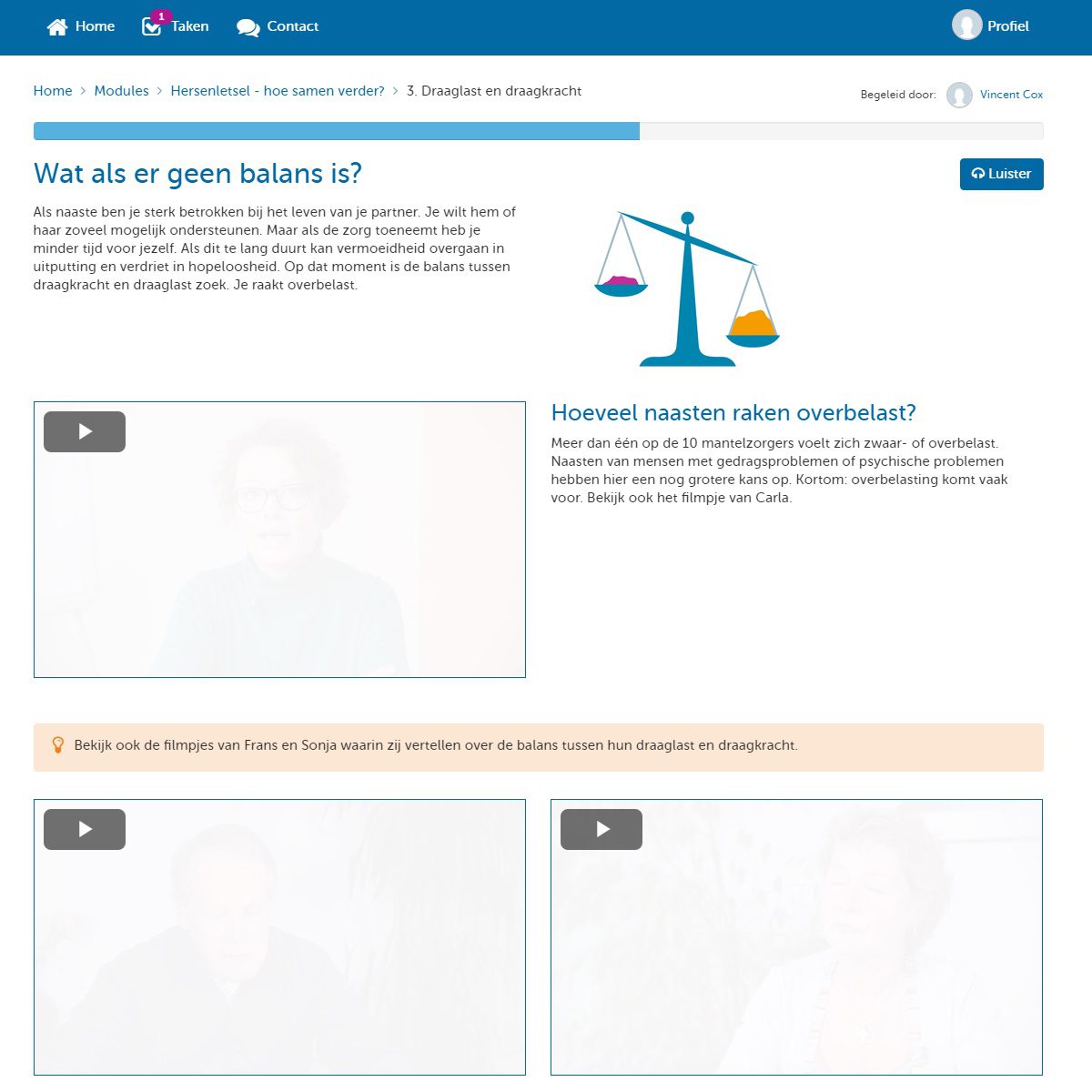

Supplement: Multimedia Appendix 2 [file resprot_v7i2e60_app2.jpg]
